# Evaluation of AlphaFold structure-based protein stability prediction on missense variations in cancer

**DOI:** 10.3389/fgene.2023.1052383

**Published:** 2023-02-21

**Authors:** Hilal Keskin Karakoyun, Şirin K. Yüksel, Ilayda Amanoglu, Lara Naserikhojasteh, Ahmet Yeşilyurt, Cengiz Yakıcıer, Emel Timuçin, Cemaliye B. Akyerli

**Affiliations:** ^1^ Department of Biochemistry and Molecular Biology, Institute of Health Sciences, Acibadem Mehmet Ali Aydinlar University, Istanbul, Türkiye; ^2^ Department of Biostatistics and Bioinformatics, Institute of Health Sciences, Acibadem Mehmet Ali Aydinlar University, Istanbul, Türkiye; ^3^ Acibadem Labgen Genetic Diagnosis Centre, Acibadem Health Group, Istanbul, Türkiye; ^4^ Acibadem Pathology Laboratories, Acibadem Health Group, Istanbul, Türkiye; ^5^ Department of Biostatistics and Medical Informatics, School of Medicine, Acibadem Mehmet Ali Aydinlar University, Istanbul, Türkiye; ^6^ Department of Medical Biology, School of Medicine, Acibadem Mehmet Ali Aydinlar University, Istanbul, Türkiye

**Keywords:** missense variants, AlphaFold, cancer, protein stability, pLDDT score

## Abstract

Identifying pathogenic missense variants in hereditary cancer is critical to the efforts of patient surveillance and risk-reduction strategies. For this purpose, many different gene panels consisting of different number and/or set of genes are available and we are particularly interested in a panel of 26 genes with a varying degree of hereditary cancer risk consisting of *ABRAXAS1, ATM, BARD1, BLM, BRCA1, BRCA2, BRIP1, CDH1, CHEK2, EPCAM, MEN1, MLH1, MRE11, MSH2, MSH6, MUTYH, NBN, PALB2, PMS2, PTEN, RAD50, RAD51C, RAD51D, STK11, TP53*, and *XRCC2.* In this study, we have compiled a collection of the missense variations reported in any of these 26 genes. More than a thousand missense variants were collected from ClinVar and the targeted screen of a breast cancer cohort of 355 patients which contributed to this set with 160 novel missense variations. We analyzed the impact of the missense variations on protein stability by five different predictors including both sequence- (SAAF2EC and MUpro) and structure-based (Maestro, mCSM, CUPSAT) predictors. For the structure-based tools, we have utilized the AlphaFold (AF2) protein structures which comprise the first structural analysis of this hereditary cancer proteins. Our results agreed with the recent benchmarks that computed the power of stability predictors in discriminating the pathogenic variants. Overall, we reported a low-to-medium-level performance for the stability predictors in discriminating pathogenic variants, except MUpro which had an AUROC of 0.534 (95% CI [0.499–0.570]). The AUROC values ranged between 0.614–0.719 for the total set and 0.596–0.682 for the set with high AF2 confidence regions. Furthermore, our findings revealed that the confidence score for a given variant in the AF2 structure could alone predict pathogenicity more robustly than any of the tested stability predictors with an AUROC of 0.852. Altogether, this study represents the first structural analysis of the 26 hereditary cancer genes underscoring 1) the thermodynamic stability predicted from AF2 structures as a moderate and 2) the confidence score of AF2 as a strong descriptor for variant pathogenicity.

## 1 Introduction

Functional impact of missense variations has been extensively studied aiming to unravel sequential and/or structural patterns that would discriminate pathogenic variants from benign variants. Apart from sequence and structure-based features, protein stability has also been shown to predict the pathogenicity of variants (Gerasimavicius et al., 2020; Sanavia et al., 2020; Birolo et al., 2021). Hitherto, many different stability predictors have been developed (Guerois et al., 2002; Bershtein et al., 2006; Kebabci et al., 2022) and reported to show promise in distinguishing pathogenic variants (Gaboriau et al., 2015; Gerasimavicius et al., 2020). Notably, the stability predictors that were reported to be successful in pathogenicity prediction were structure-based methods, i.e., they require an available structure in PDB. Because not all structures are experimentally characterized or not all parts of the polypeptide chain are resolved in a given PDB structure, we note that these predictors are restricted to the PDB availability of the variations.

AlphaFold (AF2), which is the artificial intelligence system developed by DeepMind, predicts the three-dimensional structure of a protein from its amino acid sequence (Jumper et al., 2021). Because of its high accuracy, AF2 has undeniably changed the domain of structural biology. More than 200 million AF2 computed structures have been recently deposited to the AlphaFold Protein Structure Database (AlphaFold DB, https://alphafold.ebi.ac.uk/) (Varadi et al., 2022). Furthermore, RCSB Protein Data Bank (RCSB PDB) has presented more than 1 million computed structural models including AF2 predictions (Baek et al., 2021; Burley and Berman, 2021; Jumper et al., 2021; Tunyasuvunakool et al., 2021). Evidently, AF2 offers an opportunity to close the gap between the available sequence and structure data by reshaping the structural databases and creating even larger databases for computed models (Jumper et al., 2021).

Recognizing that computed structural models are not directly derived from experimental data, we underscore that these models should be critically evaluated prior to analysis. AF2 provides multiple measures to assess the reliability of the predictions. One of these measures is calculated for each residue and thus reflects the confidence of AF2 prediction for a given amino acid position. This confidence score is called the predicted local difference distance test (pLDDT) score and is derived from the IDDT metric which is a superimposition-free measure to assess the local fit between all atoms of a model (Mariani et al., 2013). The pLDDT score measures how well the prediction matches with the available PDB data and the multiple sequence alignments (Jumper et al., 2021). Thus, we note that the availability of the residue-level confidence scores allows one to assess the quality of the computed structure for a given variant, reflecting the potential use of AF2 structures for investigating the missense variants.

Breast cancer is the most commonly diagnosed cancer worldwide with an estimated 2.3 million new cases each year. It is also the fifth leading cause of cancer mortality accounting for 6.9% of cancer deaths (Sung et al., 2021). While 75%–80% of breast cancer cases are usually sporadic, the rest of the cases are either familial (15%–20%) or hereditary (5%–10%) that are caused by germline variations in breast cancer associated genes (Fanale et al., 2020). Among these genes, Breast Cancer 1 (*BRCA1*) and Breast Cancer 2 (*BRCA2*) have been reported to have variations that increase the risk of developing breast and ovarian cancers by more than 60% basically suggesting these variations as one of the leading causes of breast and ovarian cancers (Gradishar et al., 2022). Nonetheless, the rate of non-*BRCA* pathogenic variations was higher than those of *BRCA1* or *BRCA2* pathogenic variations especially in bilateral breast cancer patients (Fanale et al., 2020). Thus, in addition to *BRCA1* and *BRCA2,* many other genes have been identified as susceptibility genes for breast cancer (Angeli et al., 2020). Among these, Partner and Localizer of BRCA2 (*PALB2*) has been reported to have pathogenic variants (Xia et al., 2006). Variations in other genes such as *PTEN* and *TP53*, which are also associated with highly penetrant syndromes, like Cowden (*PTEN*) and Li-Fraumeni (*TP53*), reported to increase breast cancer risk by 60% (Angeli et al., 2020; Peleg Hasson et al., 2020; Gradishar et al., 2022). In summary; *BRCA1*, *BRCA2, PALB2,* Serine/Threonine Kinase 11 (*STK11*)*,* Tumor Protein P53 (*TP53*)*,* Phosphatase and Tensin Homolog (*PTEN*)*,* and Cadherin 1 (*CDH1*) genes are considered as high risk genes because of their higher odds ratio than 5 while Ataxia-Telangiectasia Mutated (*ATM*)*,* BRCA1 Associated RING Domain 1 (*BARD1*)*,* Checkpoint Kinase 2 (*CHEK2*)*,* RAD51 Paralog D (*RAD51D*) and Nibrin (*NBN*) genes are classified as low-to-moderate risk genes (Peleg Hasson et al., 2020). Along with these genes, many other cell-cycle and/or DNA repair genes have been reported to have variations in breast cancer patients (Colas et al., 2019).

Identifying pathogenic variants in high-risk individuals is critical to the efforts of patient surveillance and use of risk-reduction strategies. Hereditary cancer genetic panel tests comprising different number of genes have been increasingly applied to particularly patients with a family history of cancer (Hu et al., 2020). With the advent of next-generation sequencing technologies and a parallel decline in their cost, targeted sequencing approaches, i.e., multi-gene panel tests, have been increasingly used. In addition to the advantages of targeted gene sequencing such as low cost and time efficiency compared with whole exome/genome sequencing methods, the collected data from this approach has the potential to provide insights about the mechanism of tumorigenesis broadening our knowledge on the variation landscape of a set of risk bearing genes (Chen et al., 2020). Despite these undeniable benefits, certain challenges particularly related to the counseling of patients are still present especially when the guideline information is not conclusive such as for the variations in the low penetrance genes or variations with an unknown significance (VUS).

Studies have recently pointed out the need to critically assess the risk and benefits of multi-gene panel tests (Catana et al., 2019; Reid and Pal, 2020). Especially, a high accumulation of unknown labels that may lead to patient anxiety shadow the benefits of these tests (Catana et al., 2019). More importantly, the selection of genes in the panel may not necessarily depend on their risk estimates, a situation which may result in an increase of VUS labels (Rainville and Rana, 2014). Given their earlier discovery, extensive data has been collected for the *BRCA1/2* variations while this is not the case for many genes in the panel (Fanale et al., 2020). The bias towards *BRCA1/BRCA2* is in fact aligned with the higher cancer risk associated with the variants occurring in these genes (Chen et al., 2020). Conversely, the genes with fewer number of variants such as *ATM, BARD1, CHEK2, RAD51D,* and *NBN* are classified as low/moderate risk breast cancer genes (Chen et al., 2020). Other challenges in choosing the correct panel with a correct number of genes have also been recently outlined underscoring the need for an update of the testing and communication of its results (Reid and Pal, 2020). Thus, choosing the correct panel test and more importantly choosing the correct number of genes stay an integral part of the diagnostic process.

In this study, we tested the performance of five protein stability predictors, namely, mCSM, MAESTRO, CUPSAT, SAAF2EC-SEQ, and MUpro, by using the AF2 computed structures of 26 hereditary cancer associated proteins. We initially analyzed a breast cancer cohort of 355 patients and classified the variants spotted in this cohort according to ACMG Guidelines (Richards et al., 2015). To further increase the number of missense variations, we have integrated the entire ClinVar collection (Landrum et al., 2014; Landrum et al., 2016) of missense variants in these genes. Finally, we have analyzed the structural stability of each variant in this integrated dataset by five stability predictors and assessed the power of the stability scores in pathogenicity prediction. Our results showed 1) an unbalanced distribution of the pathogenicity labels of missense variants in both the breast cancer cohort and the ClinVar set, 2) a moderate performance of the stability predictors in discriminating the pathogenic variants and 3) a novel pattern obtained from the AF2 structures with a high pathogenicity prediction power.

## 2 Materials and methods

### 2.1 Study cohort

A total of 355 breast cancer patients above the age of 18 were included in the study. Patient characteristics such as age, age of onset, sex, histological subtype, expression status of estrogen receptor (ER), progesterone receptor (PR), human epidermal growth factor receptor (HER2) were retrospectively collected. This study was approved by the Ethics Committee of Acibadem Mehmet Ali Aydinlar University in accordance with the Helsinki Declaration (Protocol No: 2020-21/07).

### 2.2 Next-generation sequencing and bioinformatics analysis

Blood samples were collected in EDTA containing tubes. Genomic DNA was isolated with QIAamp DNA Mini QIAcube kit (QIAGEN, Germany) according to the manufacturer’s instructions. DNA concentrations were measured with the QubitTM Fluorometric Quantitation system (Thermo Fisher Scientific) using Qubit HS DNA Assay kit (Thermo Scientific, US). DNA libraries were obtained using the BRCA Hereditary Cancer MASTR Plus, Multiplicom (Agilent, United States) kit. Variant screening on 26 risk carrying genes for hereditary cancers like breast, ovarian and colorectal cancer (*ABRAXAS1*, *ATM*, *BARD1*, *BLM*, *BRCA1*, *BRCA2*, *BRIP1*, *CDH1*, *CHEK2*, *EPCAM*, *MEN1*, *MLH1*, *MRE11*, *MSH2*, *MSH6*, *MUTYH*, *NBN*, *PALB2*, *PMS2*, *PTEN*, *RAD50*, *RAD51C*, *RAD51D*, *STK11*, *TP53,* and *XRCC2*) has been performed by this kit which contained five multiplex PCR primer pools. 10 ng of DNA per primer pool was used for multiplex PCR amplification, followed by barcode ligation and purification with Agentcourt AMPureXP reagent (Beckman Coulter, Beverly, MA, United States). Quantity and quality of prepared libraries were assessed by QubitTM Fluorometric Quantitation system (Thermo Fisher Scientific). For library preparation 4 ng DNA was used. After libraries were prepared, sequence analysis was performed with Illumina MiSeq instrument using MiSeq Reagent v3 kit (Illumina, US). All sequencing data were submitted to Sequence Read Archive (SRA) (https://www.ncbi.nlm.nih.gov/sra/PRJNA895859).

Bioinformatics analysis was performed using the software Sophia Genetics DDM (Sophia Genetics v4.2). GRCh37/hg19 was used as the reference genome. During variant calling, a minimum sequence coverage depth and variant fraction parameters were set to 30x and 20%, respectively. Variants were classified according to the ACMG Guidelines (Richards et al., 2015) using databases of ClinVar (Landrum et al., 2014), BRCAExchange, OMIM^®^, dbSNP (v.155), gnomAD (v2.1.1), *in silico* pathogenicity classifiers of MutationTaster (Schwarz et al., 2010), SIFT (Ng and Henikoff, 2003), PolyPhen-2 (Adzhubei et al., 2013), REVEL (Ioannidis et al., 2016). All variants with minor allele frequency (MAF2) of less than 1% in gnomAD database were considered.

### 2.3 Compilation of missense variants from ClinVar database

ClinVar database (DB) (Landrum et al., 2014) as of 10/08/2022 was queried to collect the pathogenic and benign variants observed in the 26 genes. Variants that were linked to a pathological condition containing any of the keywords “cancer”, “tumor”, “tumour” were collected. From this list, the missense variations with at least a 2-star review score were compiled as the ClinVar set. The missense variants from the current cohort were merged with the ClinVar set and the resulting list of variants were screened by using five different protein stability predictor tools (See supplementary information).

### 2.4 AlphaFold predictions

Model structures for 24 of the 26 proteins encoded by the genes under study were deposited in the webserver of AlphaFold-EBI structure database (https://alphafold.ebi.ac.uk). The structures of ATM and BRCA2 were not included in the webserver due to their larger size than 2700 amino acids (aas) but in the proteome collections. Thus, ATM and BRCA2 structures were collected from the human proteome collection (UP000005640). The structures of both proteins were predicted in sequential rounds resulting in overlapping partial structures that were labeled as F1, F2, … Fn accordingly. For instance, BRCA2 structure (3,418 aas) was predicted in twelve sequential rounds resulting in twelve overlapping structures (F1, F2, … , F12). These partial BRCA2 structures were 1,400 aas in length and had at least 1,200 aa-long overlaps with the structures preceding them in the series. The F1 structure of BRCA2 covered the amino acids between the 1st and 1400th positions, while the F2 structure covered the region encompassing the residues from the 201th to 1600th positions resulting in an overlapping prediction for the region between 201-1,400. The last prediction (F12) was 1218-aa in length and covered the final region between the positions of 2201 and 3,418. The same scheme applying to the ATM structure (3,056 aas) resulted in 10 overlapping structures. To get the full-length structures, the structures of F1, F6, and F12 for BRCA2 and F1, F5 and F10 for ATM were utilized which showed at least 200 aas overlap with each other. The overlaps were used to structurally align two sequential structures with each other and then one of the overlaps was removed. Then the separate chains were linked to each other by amide bonds generating the full-length structure for both ATM and BRCA2. Superimposition, overlap removal and model joining were performed by Chimera UCSF (Pettersen et al., 2004). During model joining, the confidence scores for AF2 predictions (pLDDT) were kept in the B-factor column of the pdb file.

### 2.5 Prediction of protein stability

Five different predictors, namely, mCSM (Pires et al., 2014), MAESTRO (Laimer et al., 2015), CUPSAT (Parthiban et al., 2006), SAAF2EC-SEQ (Li et al., 2021) and MUpro (Cheng et al., 2006), were used to predict the impact of variations on protein stability. These predictors use either sequence or structure as an input (Table 1). Among these tools, SAAF2EC-SEQ and MUpro were sequence-based predictors while mCSM, MAESTRO and CUPSAT used 3D structures as input. For structure-based methods, AF2 structures were used for all proteins. These predictors, except Maestro, compute the folding free energy change due to a mutation (ΔΔG) by subtracting the folding free energy of the mutant (ΔG_mutant_) from the folding free energy of the native form (ΔG_native_) (Kebabci et al., 2022). Maestro uses a sign convention that labels stabilizing mutations with a negative ΔΔG sign whereas other predictors label stabilizing mutations with a positive sign. For consistency, the sign of Maestro scores was reversed to label stabilizing mutations with a positive sign and destabilizing mutations with a negative sign.

**TABLE 1:** Protein stability predictors used in this study.

Name	Input	Sign convention	References
mCSM	Structure	ΔΔG< 0.0 destabilizing	Pires et al. (2014)
^a^Maestro	Structure	ΔΔG< 0.0 stabilizing	Laimer et al. (2015)
CUPSAT	Structure	ΔΔG< 0.0 destabilizing	Parthiban et al. (2006)
SAAF2EC-SEQ	Sequence	ΔΔG< 0.0 destabilizing	Li et al. (2021)
MUpro	Sequence	ΔΔG< 0.0 destabilizing	Cheng et al. (2006)

^a^ Scores from Maestro were reversed to ensure the same sign convention that produces a negative sign for stabilizing mutations and a positive sign for destabilizing mutations.

## 3 Results and discussion

### 3.1 Clinical significance distributions differed across missense and truncating variations

**FIGURE 1:**
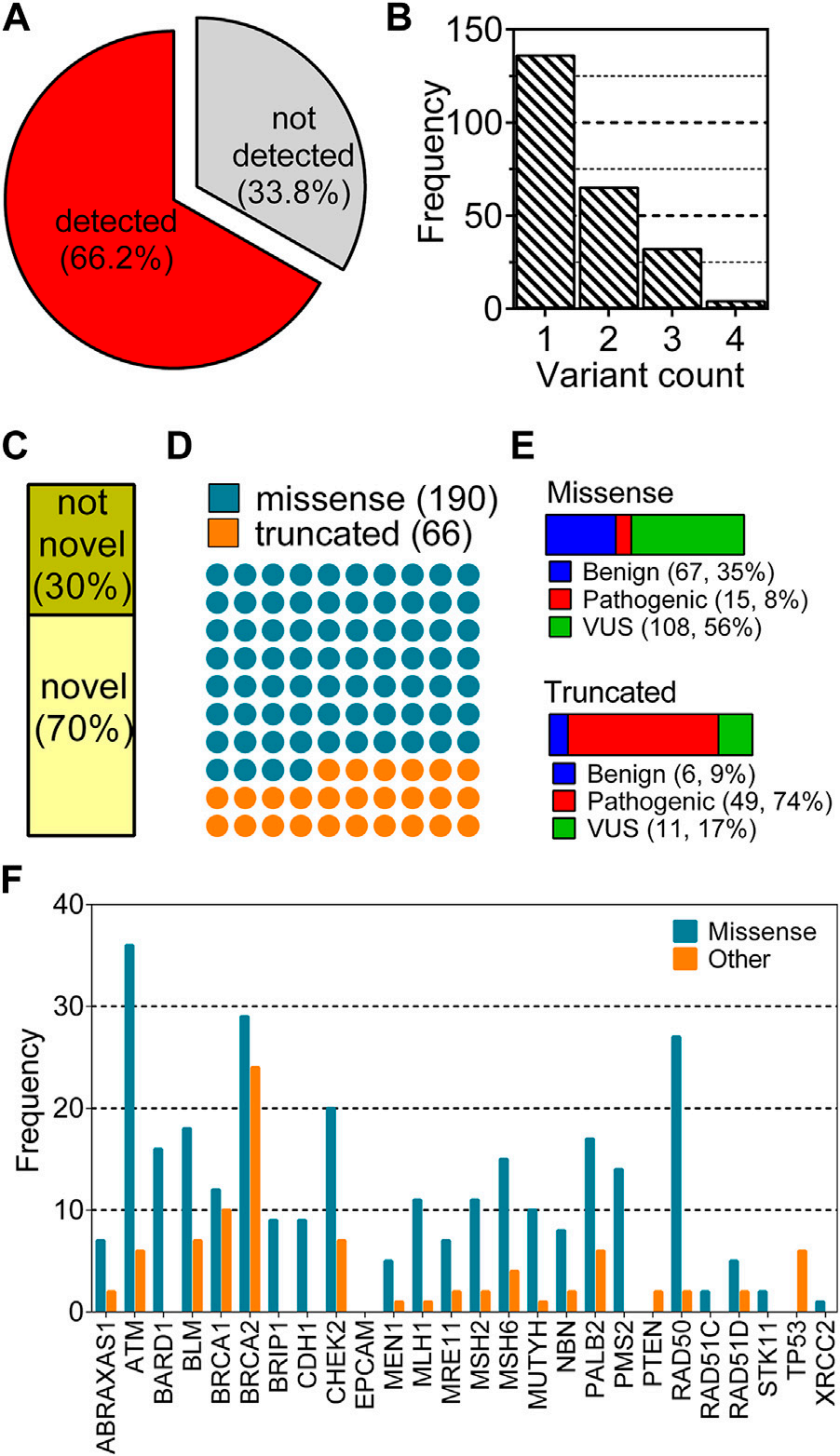
Distribution of variant characteristics collected from a cohort of 355 breast cancer patients. **(A)** shows the distributions of two groups: any type of variation detected and not detected in any of 26 genes; **(B)** shows distribution of the number of variants for each patient; **(C–F)** show the distribution of variants according to novelty, type, clinical significance and gene, respectively.

A total of 355 breast cancer patients were screened by a multigene panel of 26 cancer susceptibility genes. 237 patients (66.2%) were identified to carry at least one variation while 118 of the patients (33.8%) did not show any variations other than polymorphisms (Figure 1A). Patients that carry none of hormone receptors of ER, PR and HER2 are classified as “triple-negative,” which is an important molecular characteristic of breast cancer because of its close association with the prognosis of the disease (Brouckaert et al., 2012). The patients’ characteristics listed in Table 2 did not show different distributions between variant-detected and–not detected groups.

**TABLE 2:** Patient characteristics.

		Not detected (118)		Detected (237)			Total (355)	
		median/n	range/%	median/n	range/%	p^a^	median/n	range/%
	age	46	31–77	47	31–78	0.752	47	31–78
	age of onset	40	0–70	41	25–71	0.876	41	0–71
sex	F	118	100.0%	234	98.7%	0.554	352	99.2%
	M	0	0.0%	3	1.3%		3	0.8%
histological	Ductal	111	96.5%	219	95.2%	0.805	330	95.7%
subtypes	Lobular	4	3.5%	11	4.8%		15	4.3%
triple	No	94	80.3%	184	79.7%	0.764	278	79.9%
negative	Yes	23	19.7%	47	20.3%		70	20.1%
ER	no	40	34.2%	63	27.3%	0.237	103	29.6%
	yes	77	65.8%	168	72.7%		245	70.4%
PR	no	54	46.6%	89	38.7%	0.228	143	41.3%
	yes	62	53.4%	141	61.3%		203	58.7%
HER2	no	91	79.8%	181	80.1%	0.607	272	80.0%
	yes	23	20.2%	45	19.9%		68	20.0%
family history	no	17	15.6%	45	20.2%	0.298	62	18.5%
	yes	92	84.4%	178	79.8%		274	81.5%

^a^ Non-parametric tests were used to compare variables across variant-detected and -not detected groups. For continuous variables, Wilcoxon rank-sum test and for categorical variables Chi-square test were used.

Mostly, one variant was observed per patient, enumerating a total of 397 variations in 237 patients (Figure 1B). After the removal of duplicated variations, 256 unique variants remained for this cohort. This non-redundant set was analyzed based on the novelty (Figure 1C), the type (Figure 1D), the clinical significance (Figure 1E) and the gene of variants (Figure 1F). Of 256 unique variants, 179 have not been reported in ClinVar while the remaining 79 were found in the database. A large fraction of variants corresponding to 74% were missense variations while 26% of them were truncating type such as nonsense, frame-shift or splice site alterations (Figure 1D). Missense variants showed a dominance of VUS labels while truncated variants were mostly pathogenic (Figure 1E). Distribution of the variants across genes were also different with respect to variation types (Figure 1F). Particularly, variants of *ATM, BRCA1, BRCA2,* and *RAD50* were largely missense while *BRCAs* showed a high number of truncated variations. Some of the genes such as *EPCAM, TP53, XRCC, PTEN* and *STK11* were not reported to have any missense variations in this cohort (Figure 1F).

The collected variants from the cohort analyzed in this study reflected the dominance of VUS label in the missense variations. However, the truncated variations had mostly pathogenic labels. This observation is in line with the notion that the truncating alterations are expected to perturb the protein structure and function more than single amino acid changes (DeBoever et al., 2018). Thus, molecular understanding of the pathogenic effect of single amino acid variations is expected to be a more complex task than understanding that of truncated variations. This paradigm reflects the importance of identification of novel pattern(s) to link missense variations to any functional outcome. To this end, here we aimed to scrutinize all the variations in the 26 genes by assessing their AF2 structural stability.

### 3.2 ClinVar collection of variations from 26 genes showed unbalanced distributions

**FIGURE 2:**
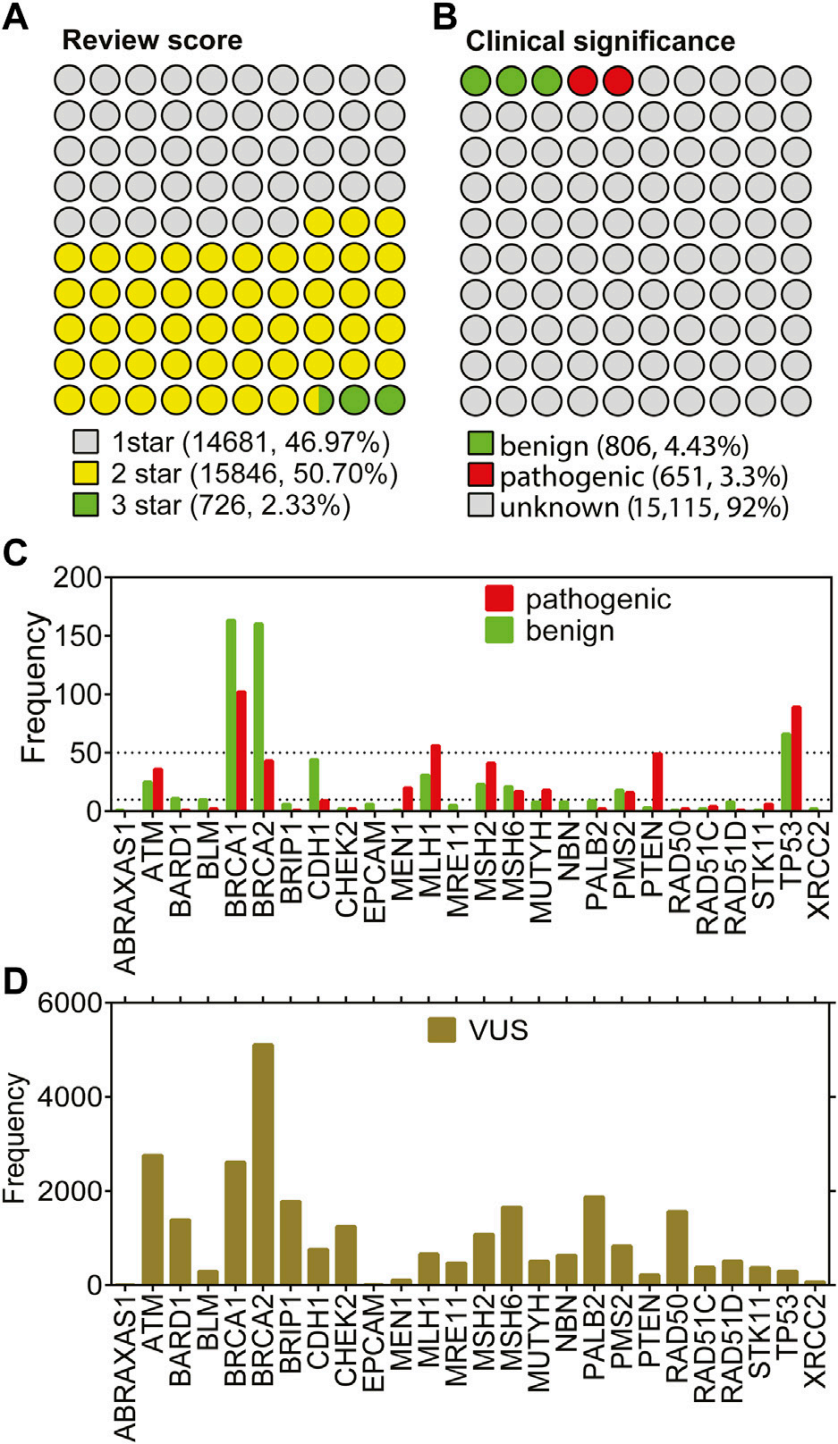
Missense variations from 26 genes in ClinVar. **(A)** shows the distributions based on annotation scores. **(B)** shows the distribution of clinical significance labels of the variants with at least 2 review scores. **(C)** shows the gene-distribution of benign and pathogenic variants from **(B)** First tick marks the frequency value of 5. **(D)** shows the distribution of VUS labels across genes.

Despite being a relatively large patient cohort, the total number of missense variations spotted by the 26-gene panel was 190. Due to 108 variants of unknown of significance (VUS), the number of missense variations with known labels were further reduced (Figure 1E-missense). To increase the number of variations in the 26 genes, all missense cancer variants that were spotted in any of the 26 genes were collected from ClinVar resulting in a total of 31,253 missense variations associated with cancer. We have eliminated the variations with conflicting labels or from single submitters (review score = 1) for the sake of the reliability, the process which almost halved the number of variants (Figure 2A). Removal of the variations with an unknown clinical significance led to a collection of variations with known labels (Figure 2B). As such only 9% (1,457/16,572) of the missense variations in ClinVar had a known clinical significance label of benign or pathogenic. This finding implied that contrary to a vast number of depositions to ClinVar DB regarding the 26 hereditary cancer genes, only a small portion (5%) could be reliably annotated with a known label. Parallel to the observation from the ClinVar collection, our variants collected from the breast cancer cohort also showed a large fraction of VUS labels in the missense group (Figure 1E). Overall, ClinVar were reported to contain 1,457 missense variations in the 26 genes of hereditary breast cancer with at least 2-star annotation scores. The final set of variants having 806 neutral and 651 pathogenic labels showed a moderately balanced distribution of the pathogenicity classes.

Figure 2C shows that the small portion with known labels was dominated by a few genes. In fact, the total number of missense variations did not add up to 10 for more than half of the genes, while it did not reach up to 50 for 19 of 26 genes. Particularly; the missense variations from the genes *ATM, BRCAs, MLH1, MSH2, PTEN,* and *TP53* were over-represented in ClinVar. Strikingly, the under-represented genes in the missense variations with either benign or pathogenic labels were also under-represented within the VUS labels (Figure 2D). For instance, missense variations from the *ABRAXAS1, BLM, EPCAM, MEN1*, and *XRCC2* genes were extremely scarce regardless of their clinical significance. The least frequent of all was ABRAXAS1 which binds to BRCA1 and form a complex essential for DNA damage response (Wang et al., 2007). Although the pathogenic/likely pathogenic variants in this *ABRAXAS1* gene are associated with an elevated risk of breast cancer (Akbari et al., 2009; Solyom et al., 2012), extremely few missense variations were observed for this protein (Figures 2D,E). We reported an unbalanced distribution of pathogenicity classes of the missense variations in ClinVar. Particularly, the known labels were much less than the unknown labels. Furthermore, the variations tended to occur in a few genes rather than having an even distribution across all 26 genes. Overall, we have collected the ClinVar variants with known labels and combined with the variants from our breast cancer cohort. The resulting set was used for the stability prediction.

### 3.3 Acquiring the full-length structures of 26 hereditary cancer proteins

To investigate the structural impact of the missense variations identified in 26 genes, we have utilized the structures predicted by AlphaFold (AF2) (Jumper et al., 2021). One reason for choosing the AF2 predicted structures over experimental ones is that the latter are not available for some of the proteins in the panel (Table 3). Second is that although some proteins have more than one experimental structures in PDB such as p53, some does not. Among these 26 proteins, only MEN1 was characterized with a full-length structure while the rest of proteins have missing and/or unmodeled regions in their structures. Notwithstanding the full-length structure advantage provided by AF2, these structures have the potential to be inaccurate or partially accurate and thus they should not be blindly accepted. To distinguish accurate predictions from inaccurate ones, we traced the per residue confidence score (pLDDT) of each variant.

**TABLE 3:** Summary of experimental and AF2 structures of 26 genes.

Gene name	Uniprot ID	Length (aa)	Number of PDB structures	AF2 prediction	Global pLDDT (median)
*ABRAXAS1*	Q6UWZ7	409	4	AF2 DB	89.01
*ATM*	Q13315	3,056	10	Human proteome	85.88^a^
*BARD1*	Q99728	777	10	AF2 DB	75.84
*BLM*	P54132	1,417	13	AF2 DB	44.02
*BRCA1*	P38398	1863	30	AF2 DB	30.66
*BRCA2*	P51587	3,418	6	Human proteome	32.615^a^
*BRIP1*	Q9BX63	1,249	3	AF2 DB	80.57
*CDH1*	P12830	882	20	AF2 DB	89.695
*CHEK2*	O96017	543	38	AF2 DB	88.74
*EPCAM*	P16422	314	2	AF2 DB	93.435
*MEN1*	O00255	615	39	AF2 DB	96.32
*MLH1*	P40692	756	7	AF2 DB	89.36
*MRE11*	P49959	708	1	AF2 DB	87.92
*MSH2*	P43246	934	9	AF2 DB	88.03
*MSH6*	P52701	1,360	7	AF2 DB	88.285
*MUTYH*	Q9UIF7	546	2	AF2 DB	92.835
*NBN*	O60934	754	2	AF2 DB	59.985
*PALB2*	Q86YC2	1,186	2	AF2 DB	37.15
*PMS2*	P54278	862	8	AF2 DB	84.82
*PTEN*	P60484	403	10	AF2 DB	95.99
*RAD50*	Q92878	1,312	1	AF2 DB	82.765
*RAD51C*	O43502	376	0	AF2 DB	92.625
*RAD51D*	O75771	328	1	AF2 DB	93.095
*STK11*	Q15831	433	3	AF2 DB	94.02
*TP53*	P04637	393	243	AF2 DB	91.36
*XRCC2*	O43543	280	0	AF2 DB	94.24

^a^ Three predictions were combined and the global pLDDT for those three structures were as follows for ATMS: 85.71, 87.455, 82.515 and for BRCA2: 31.725,28.81,83.48.

**FIGURE 3:**
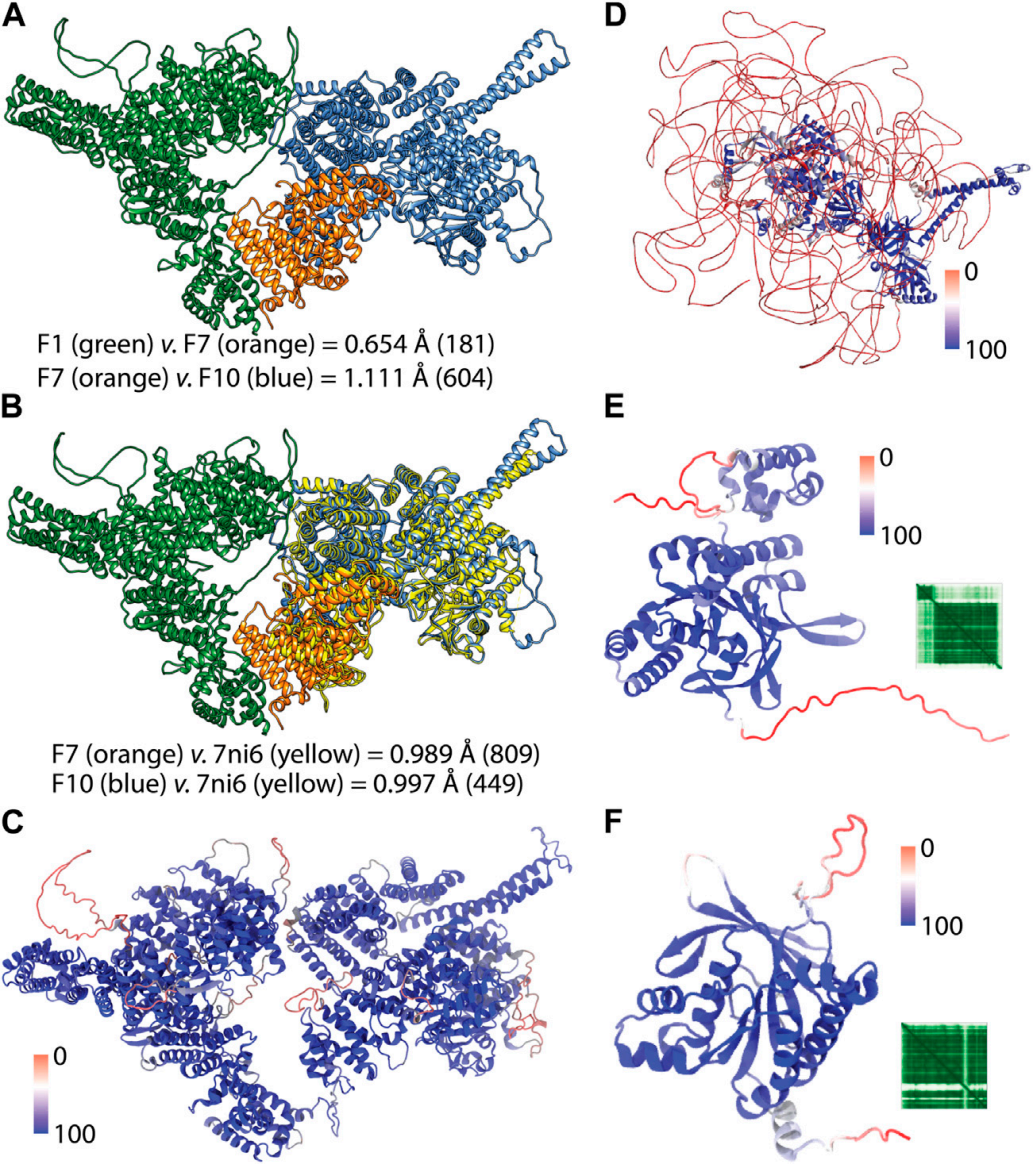
AF2 predictions of the full-length structures. **(A)** shows the pairwise superimposition of the overlapped AF2 predictions. RMSD change in the Ca trace were shown and the paired number of atoms were given in parenthesis. **(B)** shows the superimposition of the crystal structure of the C-terminus of ATM (PDB ID: 7ni6). **(C)** shows the full-length structure of ATM colored according to the confidence score pLDDT. **(D)** shows the full-length structure of BRCA2 colored based on confidence of the prediction. **(E)** and **(F)** show the AF2 structures of RAD51C and XRCC2 respectively colored according to per-residue confidence scores (pLDDT) (Jumper et al., 2021). Heatmap insets show the predicted aligned error (PAE) of the predictions which shows positional error of each residue pair (Mariani et al., 2013; Jumper et al., 2021).

Because the full-length structure of almost all human proteins can be conveniently acquired from the AF2 DB, we have utilized AF2 structures for the proteins of this gene panel (Table 3). However, we encountered a particular challenge in acquiring the full-length AF2 structures of ATM and BRCA2. These two proteins are relatively larger than other 24 proteins whose structures were readily available from the webserver of AF2 DB. On the other hand, ATM and BRCA2 structures were obtained as a part of human proteome collection (Table 3). Rather than a single structure file, more than a few structure files representing the partial overlapping structures were available for these two proteins. To acquire the full-length structure, we have iteratively superimposed the overlapping region in the structures and joined the models. For the ATM, we utilized the F1, F7 and F10 structures (Figure 3A). These structures had a high global pLDDT scores of 85.710, 87.455 and 82.515 respectively, implying that the predictions corresponding the partial fragments of ATM were accurate. By two rounds of superimposing the overlapping parts of the structures; F1 to F7 and F7 to F10; we were able to obtain the full-length structure of ATM. Particularly, F1 and F7 structures had an overlap of 200 aas and their superimposition led to a small root mean square displacement (RMSD) (Figure 3A) for the overlap region suggesting a continuum for the ATM prediction. Similarly, the superimposition of F7 and F10 structures which had a longer overlap (1,000 aas) resulted in a small RMSD change between the structures (Figure 3A). Because the structure of ATM has already been characterized (Baretic et al., 2017; Stakyte et al., 2021; Warren and Pavletich, 2022), AF2 is expected to accurately predict the ATM structure. RMSD analyses of the predicted ATM structures of F7 and F10 against the crystal structure showed well-matching coordinates (Figures 3B,C). Thus, we were able to acquire the full-length structure of ATM by iteratively aligning the overlaps in the partial structures. The resulting full-length ATM structure was confirmed to have a high global confidence score (Table 3).

For the BRCA2, iterative superimposition failed due to its extensive disorder in its structure. Although the length of the overlaps was maximized by recruiting all twelve partial structures to superimposition, the final structure was not a well-fitted one. Given the extensive disorder in the structure of BRCA2, we failed to align structures. Thus, we have predicted three non-overlapping partial structures of BRCA2 in three rounds by using ColabFold (Mirdita et al., 2022). The predicted structures were joined end-to-end to generate the full-length structure of BRCA2 (Figure 3D). We have also used the confidence score of AF2 to assess the reliability of this structure globally and locally. The full-length structure of BRCA2 led a low global confidence score (Table 3). However, when we inspected the scores for the individual predictions, we noted that the third structure corresponding to the C-terminus had a high global score implying that C-terminal region is a reliable prediction. As we colored the full-length prediction of BRCA2 structure, essentially the disordered regions were observed to have low pLDDT scores while the regions with a defined secondary structure had higher scores (Figure 3D). We also note that the AF2 predicted structures of two proteins, namely, RAD51C and XRCC2, whose structures were not experimentally studied, showed high global confidence score (Figures 3E, F).

The case of BRCA2 was a clear confirmation of why AF2 structures cannot be blindly trusted yet BRCA2 was not the sole example. For example, p53, whose structure has been extensively studied resulting in 243 experimental PDB structures (Table 3), has not been ever captured in full-length due to its intrinsically disordered poly-proline rich N-terminus (Wells et al., 2008). Furthermore, we stressed that the AF2 predicted structures of BRCA1, BLM and PALB2 had lower global pLDDT scores than 50, implying low confidence for their overall structure. In summary, AF2 predictions have certainly provided advantages, one of which is the availability of almost any human protein structure at its full-length. In our study we have utilized the confidence score of the variant positions to discriminate between the reliable and unreliable predictions.

### 3.4 Protein Stability Predictors Moderately Predicted Pathogenicity

We integrated the variant set obtained from the breast cancer cohort and ClinVar to construct the final dataset which extensively represent cancer-associated variants in the 26 genes with a known labels and with at least 2-star annotation score. For annotation of clinical significance labels, we have followed the latest ACMG Guidelines for the variants from our breast cancer variants that were not previously reported in ClinVar, (Richards et al., 2015). For the common variants that appeared both in ClinVar and our cohort, we did not report any conflicts between the ACMG guideline-based and ClinVar labels and confirmed the match between our and ClinVar labels for the common set. By eliminating the redundant variations and inconsistencies such as mismatch in the variant and Uniprot positions, 1,201 unique missense variations were collected (See supplementary information).

**FIGURE 4:**
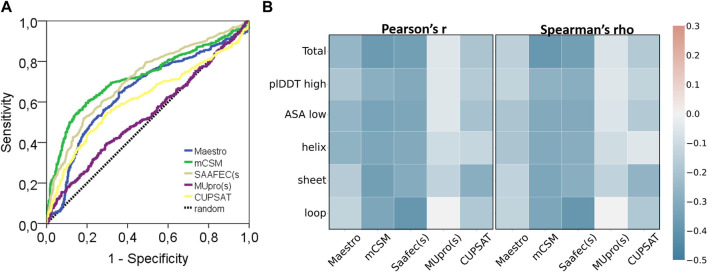
Performance of protein stability tools and two characteristics of AF2 structures. **(A)** ROC curve and **(B)** correlation analyses of ΔΔG predictors (pathogenic: 445, benign: 647).

This dataset was analyzed by five different protein stability predictors, namely, Maestro, mCSM, SAAF2EC, MUpro, and CUPSAT. Two of these predictors, SAAF2EC and MUpro utilized sequence information while the rest of the tools required three dimensional structures for which AF2 structures was recruited. We have plotted receiver operating characteristic (ROC) curves to analyze whether and how the predicted ΔΔG scores discriminate pathogenic variants from benign variants (Figure 4A). Area under ROC curve (AUROC) is a robust metric for assessment of classification performance, particularly for the skewed datasets (Jeni et al., 2013). According to AUROC calculations, mCSM and SAAF2EC that were followed by Maestro and CUPSAT showed a medium-level performance in variant classification (Table 4). Less accessible positions were included in this subset by using the relative accessible surface area (rASA) threshold value of 0.7. In other words, low pLDDT-scored regions were removed to analyze the confident regions. We noted a slight reduction in the performance of the stability predictors for these low accessible and high pLDDT-scored regions (Figures 4A, B; Table 4). Because of the close association of structural disorder and AF confidence scores (Necci et al., 2021; Ruff and Pappu, 2021), the interdomain and/or termini regions, which are likely to be disordered, are expected to have lower pLDDT scores than those of the domain regions. Thus, we consider that the second analysis involving the variants with higher pLDDT scores (Table 4) was likely to cover the variants located in the domain regions rather than the interdomain and/or termini regions.

**TABLE 4:** Area under ROC curve of scores from five stability predictors and two structural features.

	Total (P: 445, B: 647)			pLDDT≥50 (P: 410, B: 294)		
	ROC^AUC^	95% CI	*p*	ROC^AUC^	95% CI	*p*
Maestro	0.650	0.616–0.684	<0.001	0.613	0.572–0.655	<0.001
mCSM	0.719	0.687–0.752	<0.001	0.666	0.626–0.705	<0.001
SAAF2EC(s)	0.711	0.679–0.742	<0.001	0.682	0.642–0.722	<0.001
MUpro(s)	0.534	0.499–0.570	0.055	0.528	0.485–0.570	0.207
CUPSAT	0.614	0.578–0.649	<0.001	0.596	0.554–0.638	<0.001
pLDDT	0.852	0.789–0.845	<0.001	0.762	0.727–0.797	<0.001
rASA	0.817	0.830–0.874	<0.001	0.765	0.729–0.801	<0.001

Protein stability prediction is an important task contributing not only to our understanding of protein folding but also to the prioritization of variations (Gerasimavicius et al., 2020; Sanavia et al., 2020; Birolo et al., 2021). A recent study that recruited a large dataset of missense mutations which were not exclusive to cancer variants has analyzed the performance of 13 different structure-based stability predictors and reported a moderate level performance of pathogenicity prediction (Gerasimavicius et al., 2020). Particularly, the performance of the ΔΔG predictors, except MUpro, were comparable with the performance of the tools tested in this study. Another benchmark showed a higher AUROC for a stability predictor for discriminating only the MLH1 variants in Lynch syndrome (Parthiban et al., 2006). Surprisingly, the classification performance of CUPSAT and mCSM was higher on the variants of these 26 proteins (Figure 4A) than on a general dataset comprising a larger number of proteins (Gerasimavicius et al., 2020). Non-etheless, ΔΔG scores were reported to have a low-to-medium level of capacity to discriminate pathogenic variants (Figure 4A; Table 3). One plausible explanation behind a general low performance of ΔΔG prediction would be likely the alternative mechanisms driving the cancer pathogenicity other than protein destabilization/stabilization (Gerasimavicius et al., 2020). Affected protein-protein interactions (PPI) is an example of an alternative mechanism. The BRCA1 is a tumor suppressor protein that forms a multimeric complex known as the BRCA1-associated genome surveillance complex (BASC) (Wang et al., 2000). Similarly, BRCA2 protein controls the binding of the recombinase RAD51 to the DNA double-strand breaks *via* the formation of a BRCA1-PALB2-BRCA2 complex. It consists of a helical domain, three oligonucleotide binding domains, and a tower domain that allow BRCA2 to the recruitment of both single-stranded DNA and double-stranded DNA (Xia et al., 2006; Buisson et al., 2010). Furthermore, BRCA2 interacts with proteins that were coded by some of the genes in this panel such as PALB2 (Xia et al., 2006) and p53 (Marmorstein et al., 1998). PALB2 also interacts with the single-strand DNA and the recombinase RAD51D to stimulate strand invasion throughout the homologous recombination process (Angeli et al., 2020). Moreover, BRCA1 interacting protein C-terminal helicase 1 (*BRIP1*) gene encodes a protein that directly interacts with BRCT domain of BRCA1 to repair damaged DNA (Bershtein et al., 2006). This network of PPI within these proteins readily suggests that missense variations could render a pathogenic impact through affecting the complex interactions without altering the structural stability of the free form.

Another point is that, pathogenic variations were generally considered destabilizing variations. While this assertion holds for a large number of cases and also is reflected by the negative correlation between labels and ΔΔG scores (Figure 4B), some exceptions have also been covered (Tokuriki et al., 2008; Nishi et al., 2013; Stefl et al., 2013). Among these, one well-known example is the H101Q variant of CLIC2 protein which stabilizes the membrane protein in turn leading to a loss-of-function pathogenic variation (Witham et al., 2011). From this perspective, our results showing a low-to-medium level performance of ΔΔG predictions in discriminating pathogenic variants is reasonable and in agreement with the performance (Gerasimavicius et al., 2020). To reach a higher performance, we emphasize the necessity of a higher level of information for the variant positions such as their closeness to the PPI binding interfaces or the degree of flexibility/rigidity introduced by mutation. Thus, a pathogenic mutation that affects the protein-protein interactions without exerting any effect on the structural stability could be covered by the predictions (Nishi et al., 2013).

Overall, our study revealed that the stability predictors showed a similar level pathogenicity prediction performance with AF2 predicted structures compared with the performance of the predictors with the experimental structures. A recent study allocated more than 100,000 mutations and analyzed the performance difference of stability predictors with respect to the source of the structure (Akdel et al., 2022). They showed that predictions based on AF2 structures produced a comparable level accuracy to those based on experimental structures while predictions using homology models showed a substantial decrease in accuracy for the templates with low sequence identity. Thus, taken together with the results of the recent study (Akdel et al., 2022), our results have further confirmed that stability predictions based on AF structures had a comparable performance of pathogenicity classification with that based on experimental structures.

### 3.5 AF2 confidence scores affected the consistency of stability predictors

**FIGURE 5:**
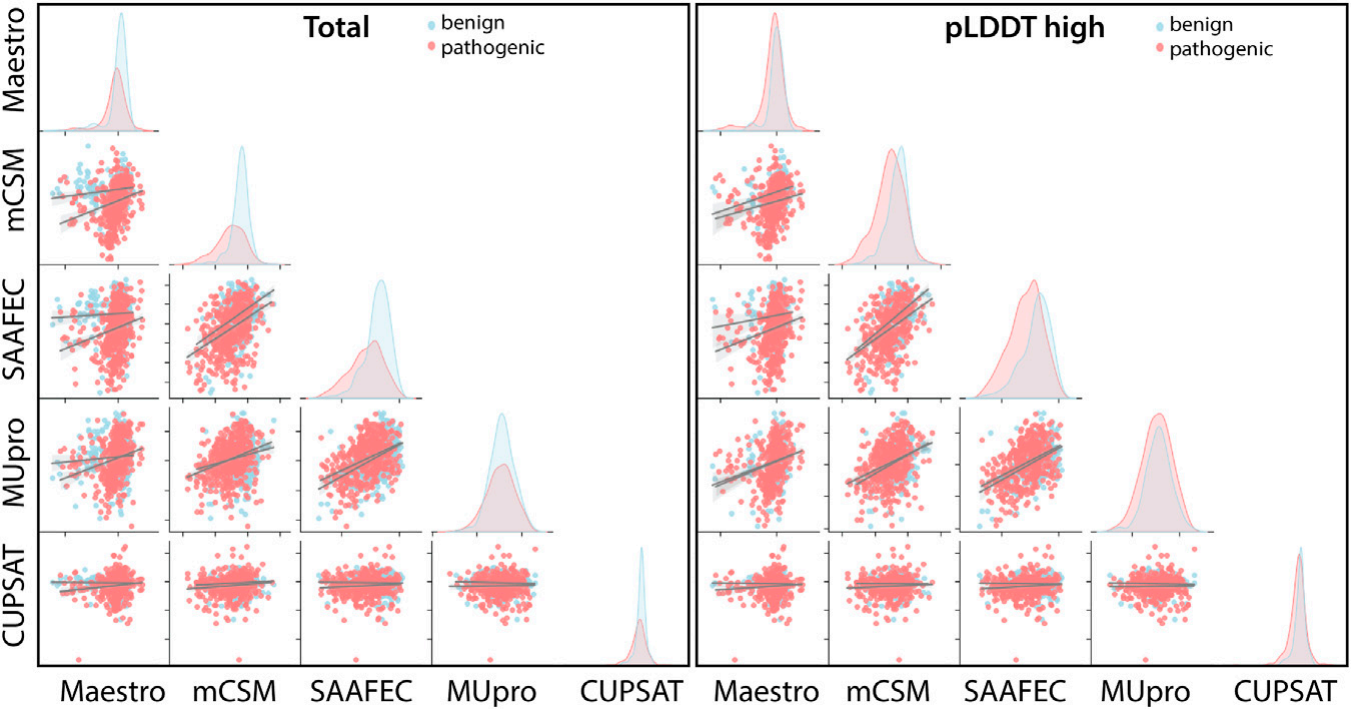
Cross-correlation of stability predictors for the total set and for the regions with high confidence (pLDDT high).

Regardless of the fact that ΔΔG predictions may not fully account for all pathogenicity mechanisms, the ΔΔG predictors are expected to produce consistent results with each other. To assess the consistency of the ΔΔG predictors, we have cross-correlated their scores for the total set as well as for the subset with high pLDDT scores (Figure 5). The cross-correlation analysis affirmed a large variation in the ΔΔG scores of different predictors. We reported a moderate level of correlation between the scores of top two performers, mCSM and SAAF2EC. On the other hand, the rest of the tools did not produce correlated scores (Figure 5). Notably, MUpro, which showed no performance in Figures 4A, B, produced correlated scores with SAAF2EC and mCSM. Contrary to the MUpro case, CUPSAT, which showed a low level of performance in discriminating the pathogenic variants, produced scores that were not correlated with any of the predictors (Figure 5). Furthermore, as we inspected the variants with higher pLDDT scores than 50, we noted a change in the cross-correlation of the Maestro’s scores for the benign variants (Figure 5). Essentially, the correlations between the scores of Maestro and the other tools were slightly improved for the benign variants with high pLDDT scores while the correlations were not affected for the pathogenic variants (Figure 5). This observation suggested that the consistency of predictions was ameliorated for the subset with high confidence scores. More importantly, because this improvement was only spotted in the benign variants but not in the pathogenic variants, this finding also implied a distinction in the pLDDT score distributions of benign *versus* pathogenic variants.

### 3.6 Benign and pathogenic variations showed distinct distributions of AF2 confidence scores

**FIGURE 6:**
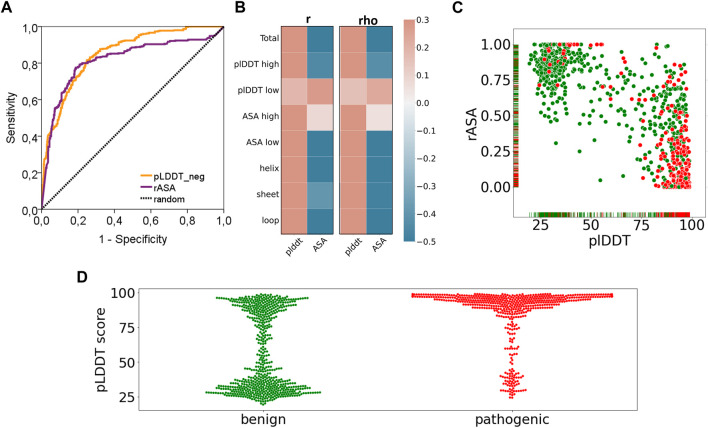
Performance of pathogenicity prediction of two characteristics of AF2 structures. **(A)** ROC curve and **(B)** correlation analyses of pLDDT and rASA values of the variants in the AF2 structures and **(C)** pLDDT vs rASA scatter plot and **(D)** pLDDT distributions (benign: green, pathogenic: red).

Finally, we have reported a surprising performance of the AF2 confidence scores in discriminating the pathogenic variants (Figure 6). Essentially, both AUC based (Figure 6A) and correlation based performances (Figure 6B) of the pLDDT scores and rASA values were reported to be good predictors for cancer pathogenicity. As the confidence score of the variation increases or the solvent accessibility of the variant position decreases, we observed a higher number of pathogenic variants (Figure 6C). This novel finding suggests that AF2 structures could be used to extract robust features such as pLDDT scores that would contribute to the future studies of machine learning models for pathogenicity prediction. The close relationship between the confidence score of AF2 predictions and pathogenicity would open new doors for one to assess the risk of missense variations.

Our results showed that pathogenic variants tend to position at locations that were more confidently predicted by AF (Figure 6C). The power of the AF confidence scores in predicting the pathogenicity of missense variants was, to some extent, unsurprising, particularly considering the close association of structural disorder and pLDDT score (Necci et al., 2021; Ruff and Pappu, 2021). Despite this partly predictable outcome, our results hold an advancement to the current literature by addressing the extent and strength of the association between variant pathogenicity and the AF confidence scores through a dataset of more than 1,000 variants. Additionally, we also reported distinct pLDDT distributions from the benign and pathogenic variants (Figure 6D). While pathogenic variants were exclusively spotted at the positions with high confidence scores, benign variants were mostly found at the positions that have either very low or very high pLDDT scores. Overall, our results revealed a partly predictable but novel link between the AF confidence scores and pathogenicity.

Recently, the power of the AF2 computed structures in predicting stability changes was analyzed by addressing the correlation between the experimental ΔΔG and the change in pLDDT scores (Pak et al., 2021; Buel and Walters, 2022). Both studies agreed on the incapacity of AF2, particularly the change in pLDDT scores upon mutation, in predicting the change in protein stability. In fact, this conclusion could be partly explained by the suggestion of AF2 developers not to use AF2 for the prediction of mutant structures. Given these studies, we note that while the confidence scores of AF2 prediction have a meaningful impact on pathogenicity prediction, the same is not true for stability predictions. Furthermore, other predictors than pLDDT scores were reported to predict variant pathogenicity by using position-specific scoring matrices (PSSMs) or structural features (Andreotti et al., 2010; Woodard et al., 2021). For the analyzed set, we reported the AF2 confidence scores showed a similar performance to these known sequential and structural features.

## Data Availability

The data presented in the study are deposited in the Sequence Read Archive (SRA), accession number PRJNA895859 (https://www.ncbi.nlm.nih.gov/sra/PRJNA895859).
